# Efficacy of Conventional and Biorational Insecticides against the Invasive Pest *Thrips parvispinus* (Thysanoptera: Thripidae) under Containment Conditions

**DOI:** 10.3390/insects15010048

**Published:** 2024-01-10

**Authors:** Livia M. S. Ataide, German Vargas, Yisell Velazquez-Hernandez, Isamar Reyes-Arauz, Paola Villamarin, Maria A. Canon, Xiangbing Yang, Simon S. Riley, Alexandra M. Revynthi

**Affiliations:** 1 Tropical Research and Education Center, University of Florida, Homestead, FL 33031, USA; yvelazquez@ufl.edu (Y.V.-H.); ireyesarauz@ufl.edu (I.R.-A.); wpaola.villamari@ufl.edu (P.V.); malejandra1@ufl.edu (M.A.C.); 2 New York State Integrated Pest Management Program, Cornell University, Portland, NY 14769, USA; gav33@cornell.edu; 3 United States Department of Agriculture, Agricultural Research Service, Subtropical Horticulture Research Station, Miami, FL 33158, USA; xiangbing.yang@usda.gov; 4 Agronomy Department and IFAS Statistical Consulting Unit, University of Florida, Gainesville, FL 32611, USA; simon.riley@ufl.edu

**Keywords:** chemical control, integrated pest management, acute toxicity, residue toxicity, mortality, feeding damage

## Abstract

**Simple Summary:**

*Thrips parvispinus* (Karny) is an invasive and polyphagous pest that attacks a wide range of plants, including ornamentals, vegetables, and fruits. Since its detection in Florida in 2020, *T. parvispinus* has emerged as a serious threat to agriculture in the United States, and it is currently a regulated pest in Florida. Immediate efforts are needed to develop strategies that can effectively safeguard crops from the detrimental impacts of this pest. With that in mind, we identified efficacious conventional and biorational insecticides that can control *T. parvispinus* infestations rapidly in ornamental plants. Larvae and adults were exposed to each insecticide either directly or through residue toxicity routes. For the direct assays, each product was applied on larvae and adults using a Potter Spray Tower. For the residue toxicity assays, each product was applied on bean plants. Larvae and adults were released after the plant tissue dried. From the 21 conventional insecticides tested against *T. parvispinus*, chlorfenapyr, sulfoxaflor-spinetoram, and spinosad caused the highest mortality and leaf-feeding damage across all stages in both assays. Among the 11 biorational insecticides tested, mineral oil (3%) and sesame oil caused the highest mortality and lowest leaf-feeding damage. We recommend implementing a rotation program that integrates these products, considering their various modes of action.

**Abstract:**

In 2020, the invasive *Thrips parvispinus* (Karny) was first detected in Florida, United States. In response to the implemented regulatory restrictions, we conducted laboratory experiments under containment conditions. Thrips larvae and adults were exposed to 32 products (conventional and biorational insecticides) either directly or indirectly. Direct exposure was performed using a Spray Potter Tower, while indirect exposure was conducted by evaluating residue toxicity against the thrips. Water served as a control. We assessed mortality and leaf-feeding damage 48 h post-treatment. Among the conventional insecticides, chlorfenapyr, sulfoxaflor-spinetoram, and spinosad caused high mortality across all stages in both direct and residue toxicity assays. Pyridalyl, acetamiprid, tolfenpyrad, cyclaniliprole-flonicamid, acephate, novaluron, abamectin, cyantraniliprole, imidacloprid, cyclaniliprole, spirotetramat, and carbaryl displayed moderate toxicity, affecting at least two stages in either exposure route. Additionally, chlorfenapyr, spinosad, sulfoxaflor-spinetoram, pyridalyl, acetamiprid, cyclaniliprole, cyclaniliprole-flonicamid, abamectin, and acephate inhibited larvae and adult’s leaf-feeding damage in both direct and residue toxicity assays. Regarding biorational insecticides, mineral oil (3%) and sesame oil caused the highest mortality and lowest leaf-feeding damage. Greenhouse evaluations of spinosad, chlorfenapyr, sulfoxaflor-spinetoram, and pyridalyl are recommended. Also, a rotation program incorporating these products, while considering different modes of action, is advised for ornamental growers to avoid resistance and to comply with regulations.

## 1. Introduction

*Thrips parvispinus* (Karny) (Thysanoptera: Thripidae) is an invasive thrips species native to Asia [1,2,3]. It is a polyphagous pest, with documented infestations on a wide array of host plants, compromising the potential yield of at least 43 different plant species from 19 families across various crop types. These include ornamental and food crops [4]. The last two decades witnessed a drastic expansion in the geographic distribution and host range of this pest. The rapid spread of *T. parvispinus* to numerous countries across five continents (Africa, Asia, Oceania, Europe, and North America) can be attributed to the increased movement of plant materials through international trade. This invasive thrips has been found to cause heavy scaring, flower drop, and upward leaf curling in many crops [5]. In regions such as India [3] and Indonesia [2], *T. parvispinus* has been reported to cause a yield loss of up to 70% in chili pepper production. In addition, it has also been responsible for significant yield reductions in greenhouse gardenia plants [6], as well as in crops like green bean, potato, strawberry, brinjal [7], and papaya [8].

Across the world, *T. parvispinus* has been designated as a pest of quarantine importance. It was detected in Florida, United States, in 2020 on Anthurium and Hoya in greenhouses [9]. Recently, *T. parvispinus* has emerged as a notable threat to Gardenia and Mandevilla production, particularly within nurseries in south Florida. The presence of *T. parvispinus* is a risk to ornamental and vegetable crops, posing a significant challenge due to the size of the horticulture and floriculture industry in the region. Florida accounts for 78% of the U.S. wholesale value of cut cultivated greens, 69% of foliage plant value, and 29% of potted flowering plant value [10]. Floriculture and horticulture play a pivotal role in Florida’s agricultural industry, concerning the market value of nursery, greenhouse, floriculture, and other commodities sold [11]. In recognition of the gravity of this threat and to prevent its further spread, the Florida Department of Agriculture and Consumer Services-Division of Plant Industry (FDACS-DPI) has implemented a quarantine. Under this regulation, nurseries found with this pest are placed under “Stop Sale and Hold Order” and not allowed to move any plant materials until the pest has been eradicated. A compliance agreement is also issued by the FDACS-DPI to these nurseries. This agreement outlines strict protocols for plant scouting, meticulous record keeping, and prudent insecticide applications. The latest updates from the EPPO, however, show that *T. parvispinus* is already present in Colorado, Georgia, North and South Carolina, and Puerto Rico [12].

Managing thrips is challenging due to their small size, cryptic lifestyle, and high population growth [13,14,15]. There are several conventional chemical insecticides registered for thrips control in nurseries or greenhouses such as azadirachtin, pyrethrins, carbaryl, acephate, spinosad, insecticidal soaps, narrow-range oil, and neem oil [16]. Depending on the species and temperature, thrips can complete their life cycle within 15–17 days [17], resulting in over 20 generations per year. When thrips populations are high and various life stages coexist, it may be necessary to apply insecticides three to five times over a span of 7–10 days [18]. Their rapid population growth also makes them prone to develop insecticide resistance [19]. Thrips resistance to insecticides has been well documented, with more than 150 reports of insecticide resistance from at least seven chemical Insecticide Resistance Action Committee (IRAC) classes for the western flower thrips (WFT), *Frankliniella occidentalis* [19]. Moreover, successful thrips control lies in the ability of insecticides to reach plant parts where thrips usually feed and hide, such as flowers [20,21]. Thereby, alternatives for pest management need to consider pest behavioral traits and different modes of action that can be integrated in a rotation program to avoid the emergence of resistance.

Despite limited knowledge regarding its biology, the life cycle of *T. parvispinus* requires 12 to 15 days from egg to adult at 27 °C [7]. Following egg hatching (~5 days), *T. parvispinus* larvae undergo two larval instars: L1 (1–2 days) and L2 (3–5 days). Subsequently, they remain in a pre-pupal stage for ~1 day and pupate for 2 days before emerging as adults [22]. Considering the current quarantine implications and the rapid expansion of *T. parvispinus*, insecticide applications are the only reliable method used for rapid suppression of this pest. Therefore, it is only a matter of time until insecticide-resistant thrips populations appear. The problem worsens due to the polyphagous nature of the pest, increasing the risk of infestation of other host plants. Taken all together, the presence of *T. parvispinus* raises significant concerns due to its potential to spread, particularly outside quarantined areas in southern Florida. 

The identification of insecticide products with reduced non-target effects that can be used in rotation programs for the management of *T. parvispinus* should be a priority. The use of conventional chemicals such as neonicotinoids has decreased significantly due to the detrimental effects reported on pollinating insects [23,24]. Biorationals are those insecticides that pose minimal risk to beneficial organisms, as long as direct contact between the product and the arthropod is avoided [25]. Examples include plant-derived essential oils or highly refined petroleum oils (including mineral oils). Plant-derived essential oils, also called horticultural oils, can repel or kill insects, with mortality resulting from either asphyxiation due to spiracle blockage or poisoning caused by alterations in metabolic processes [26,27]. The efficacy of horticultural oils such as *Thymus vulgaris* (thyme) and *Satureja montana* (winter savory) have proved to be highly repellent to WFT [28], while *Mentha pulegium* (mentha) and *Nepeta cataria* (catnip) showed lethal and sublethal effects [29]. To our knowledge, the efficacy of horticultural oils against *T. parvispinus* has not been investigated. 

The limited introduction of new insecticides for thrips control in recent decades underscores the significance of biorational insecticides, which include horticultural oils, and insecticidal soaps as valuable resources for managing thrips effectively. To better manage *T. parvispinus*, a strategic rotation of conventional and biorational insecticides will enhance thrips control while also mitigating the potential for resistance development [19,30]. With that in mind, and in light of the regulatory restrictions in place, we aim to identify efficacious conventional and biorational insecticides that can offer rapid control of *T. parvispinus* infestations in ornamental plants and define the most viable insecticides to integrate into a rotation plan. To achieve this goal, we evaluated 32 different products against *T. parvispinus* in a containment facility under an FDACS-DPI permit. Twenty-one conventional insecticides and 11 biorationals were tested. Contact toxicity experiments were carried out using two exposure routes: (i) direct application of these products on larvae and adults using a Potter Spray Tower, and (ii) indirect exposure by evaluating residue toxicity through the application of these products on bean plants before offering them to both larvae and adults. The efficacy of these products was assessed through thrips mortality rates and leaf-feeding damage.

## 2. Materials and Methods

### 2.1. Plant Material

Bean plants (*Phaseolus vulgaris* L. var. Roman) (Goya Foods^®^, Jersey City, NJ, USA) and pepper plants (*Capsicum annuum* var. Pepper Mini Bell Red Organic) (Harris Seeds^®^, Rochester, NY, USA) were grown from seeds. Weekly sowings of bean seeds were conducted in 4 ½ oz. pots, while pepper seeds were planted in 1-gallon pots, using ProMix soil (ProMix BX Mycorhizae, Denver, CO, USA). For germination, the pots were placed in a climate-controlled room at 25 ± 2 °C, RH 50%, and a 12:12 h (L:D) photoperiod. Plants received regular watering three times a week. Five-week-old pepper plants and two-week-old bean plants were used as a food source to maintain the *T. parvispinus* colony. All experiments were performed using two-week-old bean plants. 

### 2.2. Thrips Parvispinus Colony

The *T. parvispinus* colony was established in the containment facility of the Tropical Research and Educational Center (TREC) in Homestead, Florida (25.50° N, 80.49° W) under permit #2022-105. This colony originated from individual thrips collected from Mandevilla plant samples (*Mandevilla* sp.) that were submitted to the Plant Diagnostic Clinic for examination. The identification of the specimens was confirmed by the FDCAS-DPI, and since then, the colony of *T. parvispinus* has been maintained at 27 ± 1 °C, RH 70%, 12:12 h (L:D) in a Panasonic growth chamber (Panasonic Versatile Environmental Test Chamber MLR-352H). The colony itself was enclosed within an insect-proof mesh cage (47.5 × 47.5 × 93.0 cm, mesh diameter 80 μm) inside the growth chamber. To maintain the colony’s population, fresh bean plants were introduced into the cage three times a week. Since pepper is a preferred host [4,9], once per week a pepper plant was also added to enhance the thrips population. In addition to fresh plants, pollen from *Typha* spp. (Biobest^®^, Westerlo, Belgium) was added using a brush to supplement their diet. Every two weeks the colony underwent a refreshment process. This involved removing old and dried plants from the cage and subjecting all waste material from the colony to autoclaving before disposal.

Prior to the experiments, larval cohorts were created by transferring adult females and males from the stock colony into large Petri dishes (d = 135 mm). These Petri dishes were filled with a layer of moist cotton wool (Fisherbrand^®^, Pittsburgh, PA, USA) and a bean leaf with the abaxial surface facing up. Using a manual aspirator, adults were transferred to the Petri dishes which were immediately closed with lids adapted with a fine mesh to ensure ventilation. During a 24 h period, the adults were allowed to mate and lay eggs. Then, adults were removed and the Petri dishes containing only eggs were placed in a Panasonic growth chamber at 27 ± 1 °C, RH 70%, 12:12 h (L:D). First-instar larvae (L1, 5 days old), second-instar larvae (L2, 6 days old), and adults (females at random ages) were used in the experiments.

### 2.3. Residue Toxicity Assays

Insecticide solutions were prepared following the maximum label rates for thrips (whenever possible) (Table 1), with 1000 mL of these solutions loaded into HDX^TM^ (Root-Lowell Manufacturing Co., Lowell, MI, USA) 1.7 L. handheld sprayers. Two-week-old bean plants were randomly selected and assigned to one of the treatments. Each plant was sprayed until runoff and allowed to dry for approximately four hours at room temperature until the leaves were completely dry. Water was included as a control. From each treated plant, leaves were collected, and leaf discs (d = 24 mm) were prepared using a Fisherbrand^®^ cork borer. Each Petri dish (d = 5 cm) received one leaf disc, positioned with the abaxial surface facing downward. Leaf discs were placed on water saturated cotton wool (for larval testing) or agar (for adult testing). Cotton wool was used for larvae to prevent them from escaping, while agar (1% Difco^TM^ Agar Bacteriological, Winsor & Newton, London, UK) was used for adults to prevent them from drowning. Five individuals from each developmental stage (L1, L2, and adult) were transferred using a fine paint brush (Cotman^TM^, Thermo Fisher Scientific Inc., Waltham, MA, USA) to each Petri dish. To reduce their mobility before transferring them to the leaf discs, adults were first placed into large Petri dishes (d = 135 mm) and kept in contact with ice packs for ~10 min. Following the transfer of thrips into the Petri dishes, the dishes were closed using modified lids with a fine mesh to facilitate ventilation. Each Petri dish represented one replicate, and a total of ten replicates were prepared for each treatment and developmental stage. Petri dishes were placed into a Panasonic growth chamber (27 ± 1 °C, RH 70%, and 12:12 h L:D photoperiod). Mortality assessments were performed 48 h post-treatment by recording the number of individuals that were alive, dead, or missing. This time frame for evaluations was implemented considering the quarantine re-inspection option offered by the FDACS-DPI inspectors after 48 h, and that senescence of leaf discs after 48 h could be affecting insect survival and feeding behavior. Additionally, feeding activity was also evaluated for all stages 48 h after the treatment. The leaf-feeding damage was quantified by calculating the proportion of leaf disc (d = 24 mm) area damaged (scarred) by the thrips using the Image J software version 1.54t [31]. Products were evaluated in a series of groups (blocks in time) of ten replicates, and in each group, a water control was included.

### 2.4. Direct Contact Assays

The experimental design for direct contact assays followed the same procedure as the residue toxicity assays evaluating the same insecticides (Table 1), with the only distinction being the application method, where in this case, a Potter Spray Tower (Burkard Manufacturing Co., Ltd., Rickmansworth, UK) was used. The spray tower directly applied 0.8 mL of product (5 mg/mm^2^) at a pressure of 5 PSI to each leaf disc with 5 thrips larvae (L1; L2) or 5 adults. After each application, any remaining product was removed by drying it with paper towels. Following each treatment, the sprayer underwent a cleaning process, which included the application of 1 mL of pure acetone, 1 mL of pure ethanol, and 1 mL of distilled water. After the cleaning, the sprayer was thoroughly dried, and we proceeded with the application of the next treatment. Consistently with the residue toxicity assays, ten replicates were performed for each treatment and developmental stage. Mortality assessments and leaf-feeding damage evaluations were performed 48 h post-treatment, following the same approach used in the residue toxicity assays. Products were evaluated in a series of groups (blocks in time) of ten replicates, and in each group, a water control was included.

### 2.5. Statistical Analyses

Data analysis was performed in R version 4.1.3 [32,33]. The data on the proportion of thrips mortality and the percentage of leaf damage at 48 h post-treatment were fit to separate generalized linear mixed models (GLMM) implemented with the *glmmTMB* package [34], with the two dependent variables treated as binomial and beta distributed, respectively [35,36]. In both cases, the logit link function was employed, and the data were transformed prior to analysis in order to meet the assumptions of the beta (ibid) and binomial [37,38] models.

The models included as fixed effects the following: ‘treatments’ (32 products and water control), ‘thrips developmental stages’ (including L1, L2, and adults), and ‘exposure route’ (direct and residue toxicity assays), along with all higher order interactions. The random effects, reflecting the underlying experimental design, included “trial” (the experimental set of ten treatments evaluated independently) and the blocks in time nested within trials. 

Following model fitting, F-tests were performed, and on the basis of those results, marginal mean proportion of thrips killed and percentage of leaf damage were estimated [39] for each combination of stage and application method. Within each of these combinations of stage and application methods, the efficacy of each of the treatments was compared to the control, with the results being reported in terms of the odds ratio and *p*-values adjusted using Dunnett’s procedure. In the case of thrips mortality, the odds ratio reflects the relative chance of thrips survival compared to those exposed to the control. An odds ratio of 1 indicates that an individual insect (of a given developmental stage being controlled using a given application method) is equally likely to die following exposure to either treatment or control, while an odds ratio greater than 1 indicates that the relative chance of survival is reduced by exposure to the treatment. In the case of leaf-feeding damage, the “odds ratio” reflects the proportion of damaged leaf compared to leaf discs treated with water (control). Thus, a leaf-feeding damage “odds ratio” (hereafter referred to as “relative damage”) of 0.5 indicates that treated leaf discs exhibit, on average, half as much damage as those treated with the control.

## 3. Results

### 3.1. Effect of Conventional and Biorational Insecticides on Thrips Mortality

**Figure 1:**
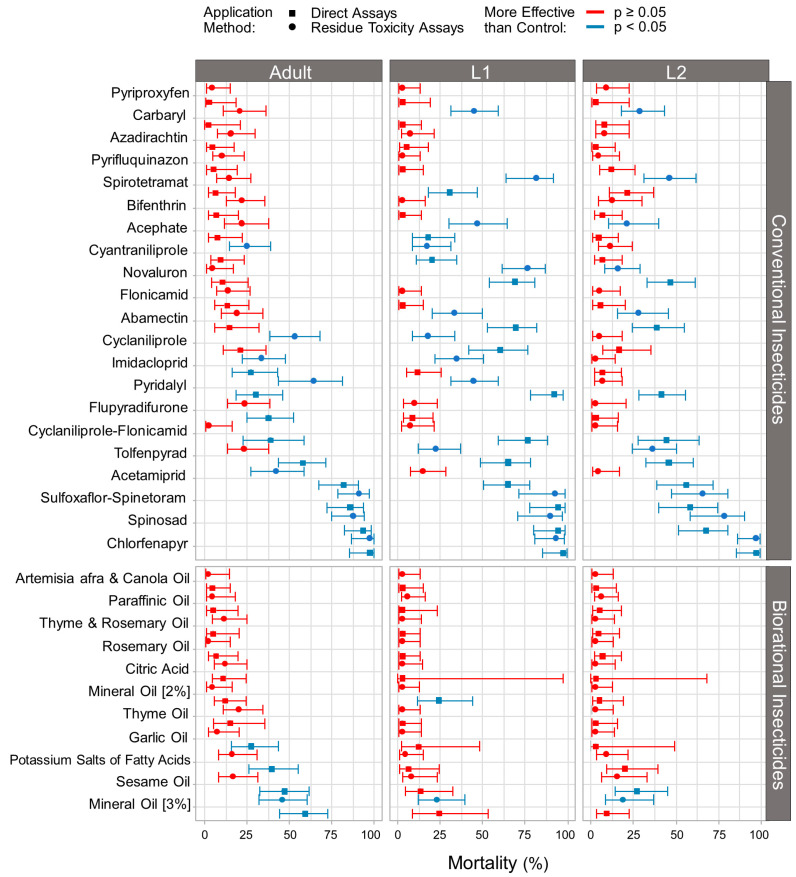
Mortality caused by conventional and biorational insecticides 48 h after treating *Thrips parvispinus*. The figure illustrates the percentage of dead larvae (L1; L2) and adult thrips in both direct (squares) and residue toxicity assays (circles). Blue color indicates that the observed mortality was significantly higher than the control (*p* ≤ 0.05; GLMM), while red indicates non-significant differences.

Direct and residue toxicity exposure to conventional and biorational insecticides caused significant larval (L1, L2) and adult thrips mortality 48 h post-treatment (GLMM: Wald χ^2^ = 1147.3; d.f. = 32; *p* ≤ 0.001, Figure 1). Moreover, there was a significant interaction among treatments and thrips developmental stages (GLMM: Wald χ^2^ = 275.8; d.f. = 64; *p* ≤ 0.001); treatments and application methods (GLMM: Wald χ^2^ = 180.9; d.f. = 32; *p* ≤ 0.001); and treatments, developmental stages, and application methods (GLMM: Wald χ^2^ = 99.1; d.f. = 64; *p* = 0.003). 

Among the conventional insecticides, in contrast to the control group, chlorfenapyr, sulfoxaflor-spinetoram, and spinosad caused the highest mortality rates among all thrips stages in both direct and residue toxicity assays (Figure 1). Acetamiprid, tolfenpyrad, pyridalyl, cyclaniliprole-flonicamid, acephate, novaluron, abamectin, cyantraniliprole, imidacloprid, cyclaniliprole, and spirotetramat were moderately toxic, significantly affecting larvae or adults in direct or residue toxicity assays. Carbaryl caused mortality of thrips larvae only during the residue toxicity assays. Pyriproxyfen, azadirachtin, pyrifluquinazon, bifenthrin, and flonicamid were found to be the least lethal, as they did not cause significant mortality in any stage in either exposure route when compared to the control group. Among the biorational insecticides tested, in contrast to the control group, mineral oil (3%) caused the highest mortality rates in all thrips stages in residue toxicity assays. Sesame oil, mineral oil (2%), potassium salts of fatty acids, and garlic oil were moderately toxic, affecting at least one thrips developmental stage in direct or residue toxicity assays.

### 3.2. Effect of Conventional and Biorational Insecticides on Thrips Leaf-Feeding Damage

**Figure 2:**
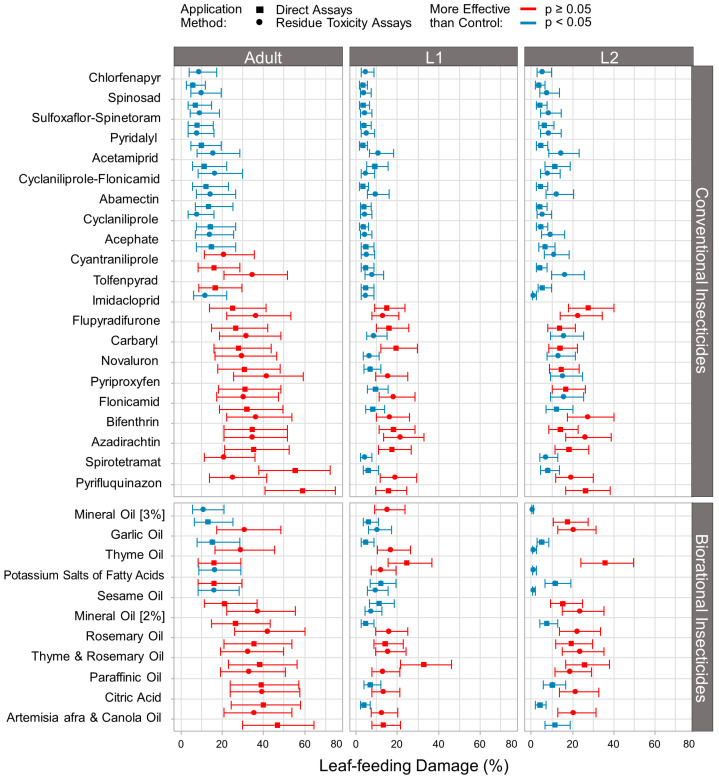
Percent of leaf-feeding damage caused by *Thrips parvispinus* 48 h after being treated with conventional and biorational insecticides. The figure illustrates the percentage of bean leaf-feeding damaged area caused by larvae (L1; L2) and adult thrips in both direct (squares) and residue toxicity assays (circles). Blue color indicates that the observed leaf-feeding damage was significantly higher than the control (*p* ≤ 0.05; GLMM), while red indicates non-significant differences.

Direct and residue toxicity exposure to conventional and biorational insecticides promoted significant differences in the proportion of leaf-feeding damage caused by larvae (L1; L2) and adult thrips 48 h post-treatment (GLMM: Wald χ^2^ = 2364.0; d.f. = 32; *p* ≤ 0.001, Figure 2). In addition, there was a significant interaction among treatments and thrips developmental stages (GLMM: Wald χ^2^ = 427.0; d.f. = 64; *p* ≤ 0.001); treatments and application methods (GLMM: Wald χ^2^ = 365.2; d.f. = 32; *p* ≤ 0.001); and treatments, developmental stages, and application methods (GLMM: Wald χ^2^ = 732.5; d.f. = 64; *p* ≤ 0.001).

Among the conventional insecticides, chlorfenapyr, spinosad, sulfoxaflor-spinetoram, pyridalyl, acetamiprid, cyclaniliprole, cyclaniliprole-flonicamid, abamectin, and acephate were effective in reducing both L1 and L2 and adult leaf-feeding damage in both exposure routes when compared to the control group (Figure 2). Carbaryl reduced leaf-feeding damage of thrips larvae only in the residue toxicity assays. Among the biorational insecticides tested, mineral oil (3%), sesame oil, garlic oil, and the insecticidal soap were the most effective in reducing leaf-feeding damage of all thrips stages in both direct and residue toxicity assays. Rosemary oil, thyme oil, thyme and rosemary oil, and artemisia afra and canola oil were the least efficient oils in preventing thrips feeding activity. While thyme and artemisia afra and canola oil significantly affected leaf-feeding damage of second-instar larvae in both direct and residue toxicity assays, they did not reduce first-instar larvae and adult thrips leaf-feeding damage and caused low mortality. 

Overall, the treatments that caused high mortality and reduced leaf-feeding damage in all thrips stages and exposure routes were chlorfenapyr, sulfoxaflor-spinetoram, and spinosad. Pyridalyl also proved to be an effective treatment against *T. parvispinus* as it successfully reduced thrips leaf-feeding damage, although mortality was not different from that of the control in L2 when tested in the residue toxicity assays. 

## 4. Discussion

In this study, 32 conventional and biorational insecticides were tested under laboratory conditions for their acute toxicity on *T. parvispinus* in a containment facility at TREC. Our evaluations focused on mortality rates and the impact of these products on the feeding activity of both larval and adult stages of the thrips. Seventeen different IRAC modes of action groups were represented among the insecticides tested [40]. This study, conducted in a quarantine facility, aimed to investigate two routes of exposure, a direct and an indirect (residue toxicity). It assessed whether, beyond immediate toxicity upon direct exposure, products also exhibit residual effects that hinder pest colonization after application. Our observations consistently revealed that products demonstrating high mortality rates under direct contact conditions also display residual effects. The only exception was carbaryl (1A), which only exhibited residual toxicity without demonstrating direct toxicity within the 48 h experimental period. Four conventional insecticides, spinosad, chlorfenapyr, sulfoxaflor-spinetoram, and pyridalyl, along with two biorationals, 3% mineral oil and sesame oil, are the most promising choices for effective control of *T. parvispinus*. Due to quarantine regulations and the requirements set by the FDACS-DPI in response to this pest, conducting long-term experiments and greenhouse testing for the efficacy of these products was not permitted at this moment. However, this work provides novel consistent information regarding effective modes of action that can be implemented as a first front of defense against an invasive pest that is currently expanding its range [12]. 

Among all of the tested products, three conventional insecticides consistently excelled in all assays. Spinosad, chlorfenapyr, and sulfoxaflor-spinetoram caused the highest mortality rates for all developmental stages of *T. parvispinus* and effectively suppressed larval and adult feeding in both exposure routes (Figure 1). Supporting our findings, spinosad has been previously shown to be highly effective against *T. parvispinus*, reducing 80% of its incidence in chrysanthemum plants in the field [41]. Additional active ingredients that have been found to decrease *T. parvispinus* populations in the field through consecutive applications include methomyl (1A), butenolides (4D), spinetoram (5), novaluron (15), and tolfenpyrad (21A) [42]. Except for the two aforementioned studies, there is no other research evidence on insecticide efficacy against this thrips species.

Spinosad is a spinosyn (5) initially introduced in 1998 and is widely used by greenhouse producers in the USA to combat WFT. It initially provided excellent thrips control [43,44], but in recent years, its efficacy against WFT has been decreased, likely due to the development of resistance [45,46]. Spinosad is derived from the Actinomycete bacterium *Saccharopolyspora spinosa* and acts through its metabolites, spinosyns A and D [47]. It has both contact and ingestion activity, with a rapid killing effect within one to three days and up to two weeks of residual activity [48]. Spinosad exhibits translaminar movement within leaf tissue. This quality enhances its efficacy in controlling pests like thrips that feed on the undersides of leaves, which can be difficult to target with contact insecticides alone. Nonetheless, it is worth noting that spinosad has been found to exhibit toxicity to natural enemies in topical toxicity tests and residual tests [49]. As a precaution, we advise its inclusion in rotation schemes with other products such as chlorfenapyr, pyridalyl, and horticultural oils for effective control of *T. parvispinus*. It is also important to schedule spinosad applications in relation to its compatibility with different natural enemies used in biological control programs implemented either in the nurseries, greenhouses, or the different crop systems [50,51].

Chlorfenapyr belongs to the chemical class of pyrrole insecticides (13). It acts as a pro-insecticide, meaning it is converted into its active form within the target pest’s body. Since it operates through a different mode of action compared to other insecticides commonly used against thrips, it makes it less susceptible to resistance development, even though resistance in two-spotted spider mites (*Tetranychus urticae*; Acari: Tetranychidae) has been documented [52]. This unique mode of action can help in rotating chemical control methods to prevent the buildup of resistant pest populations. Chlorfenapyr is known for its extended residual activity. Once applied, it can continue to control thrips and other pests for an extended period, making it an effective choice for integrated pest management programs in greenhouse, as it is currently labeled in Florida. In fact, other thrips species such as the WFT and *Frankliniella intonsa* have been highly susceptible to chlorfenapyr, resulting in 100% mortality within 12 h post-treatment by either direct spray, exposure to residue, or oral ingestion [53]. 

Sulfoxaflor-spinetoram has been used to control chewing and sap-feeding insects such as thrips, aphids, whiteflies, mealybugs, caterpillars, leaf-feeding beetles, and scales in ornamentals [54]. Sulfoxaflor is a sulfoximine (4C), a newer insecticide class that was first registered by the EPA in 2013. It is relatively volatile, and due to its rapid degradation rate, it greatly reduces the risk to beneficials [55]. Recently, it was demonstrated that the sulfoxaflor-spinetoram combination is highly effective against the hibiscus bud weevil, *Anthonomus testaceosquamosus* [56]. Likewise, this product proved to be highly effective against *T. parvispinus*. Nevertheless, further comprehensive experiments are required to validate its efficacy in commercial settings. Its use is also recommended to pests that are becoming resistant to carbamate, neonicotinoid, organophosphate, and pyrethroids [57].

Pyridalyl, a recently discovered insecticide of a novel chemical class (IRAC unclassified insecticide) was highly effective against *T. parvispinus*. It has been proved to be an effective and selective product exhibiting high insecticidal activity against Lepidoptera and Thysanoptera [58,59]. It caused high mortality of all developmental stages (second- to sixth-instar larvae) of *Plutella xylostella*, *Heliothis virescens* [60] and *Spodoptera litura* [61]. This compound was also highly toxic to second-instar larvae and adult WFT under direct spray trials [59]. In addition, no acute toxicity of this product was observed on the beneficial insect and thrips predator *Orius stringicollis* and the pollinating insect *Bombus terrestris* [59]. Pyridalyl also showed low toxicity to many other beneficial and non-target insects such as *Amblyseius cucumeris*, *Tetragnata praedonia*, *Apis mellifera*, and *Harmonia axyridis* [59] and to the natural enemies *Coleomegilla maculata*, *Hippodamia convergens*, *Geocoris punctipes*, *Bracon mellitor*, *Cardiochiles nigriceps*, and *Cotesia marginiventris* in laboratory and field experiments [49]. There are no reports on cross-resistance between pyridalyl with other currently used insecticides [60].

The diamides cyclaniliprole and cyantraniliprole along with the cyantraniliprole-flonicamid combination (28–29) were anticipated to exhibit high residual toxicity to *T. parvispinus*. However, they only demonstrated moderate toxicity. Diamide (28) insecticides are known to have long residual efficacy against a broad spectrum of chewing and sap-sucking pests on several plant species [62,63]. They provide comparable pest protection to neonicotinoids but with reduced toxicity toward beneficial arthropods, mammals, and pollinators [64]. Given the limited evaluation period in our experiments, a recommendation is made to assess the residual toxicity of these products against larval and adult stages beyond the initial 48 h period. Nevertheless, in the current experiments, feeding damage caused by all thrips stages was significantly less when cyclaniliprole and cyantraniliprole-flonicamid were applied in both direct and residue toxicity assays (Figure 2).

Our study emphasizes the use of natural products due to their advantages over synthetic insecticides. Biorational insecticides provide eco-friendly solutions for managing pest and disease control [65,66]. These natural options are known for their efficacy against a wide spectrum of insect pests while being harmless to beneficial insects [67]. They encompass mineral-based options like sulfur and kaolin-based products which are effective against various insects and foliar diseases. While the IRAC groups for biorational insecticides are yet to be classified, it is known that mineral and paraffinic oils function through mechanical and physical disruption. For instance, thyme and rosemary oils are recognized for their repellent, suffocant, endocrine disruption and neurotransmitter interference properties [65,68,69]. In our study, these two compounds had low efficacy against *T. parvispinus*. Instead, 3% mineral oil and sesame oil emerged as the most potent agents, displaying significant efficacy in inducing mortality and suppressing feeding activity in *T. parvispinus*. Furthermore, potassium salts of fatty acids and garlic oil proved effective in reducing leaf-feeding damage. In the case of garlic oil, it has been proven that it has potent spatial repellency against *Aedes aegypti* adult mosquitoes, in medical entomology and in combating the transmission of tropical viral diseases [70]. Among the low-toxic synthetics that we tested, potassium salt fatty acids, a type of insecticidal soap, is known for exhibiting dual functionality, serving as an insecticide and herbicide, effectively targeting soft-bodied insects and weeds when directly applied. However, soaps can be phytotoxic to certain crops and detrimental to beneficial insects [71,72]. Hence, it is essential to conduct further comprehensive experiments to confirm its efficacy under commercial environments. 

Controlling thrips with insecticides can be challenging due to several factors, including their cryptic lifestyle, high mobility, the feeding behavior of both larvae and adults, high reproductive rates, short generation time, insecticide resistance, and more [13,14,15]. Improper timing of application and inadequate spray coverage when using contact insecticides are common mistakes that can potentially prevent efficacious insecticides from providing control. It is widely recognized that biorational insecticides have significant potential in the management of insect pests, as they are capable of either killing or deterring a wide range of insect-feeding pests, including thrips [65,66]. In addition to their various modes of action, another advantage is their biodegradability, since they naturally break down and do not linger in the environment. These alternatives offer more environmentally sustainable approaches to managing pests and diseases. Rotating conventional and biorational insecticides with different modes of action within the thrips management program will not only lower the selection pressure for insecticide resistance development but will also prolong the efficacy of currently available insecticides [30,73]. Hence, the inclusion of biorational insecticides into rotation schemes is recommended, and their contribution to sustainable agriculture seems promising, provided that applications adhere to the label rate and registered application site (whether it is a nursery, greenhouse, or landscape).

The management of *T. parvispinus* must focus on several key areas. These encompass the exploration of eco-friendly alternatives like biorational insecticides and insect growth regulators, the development of effective strategies for managing resistance, the incorporation of precision agriculture technologies to reduce insecticide usage, and the investigation of biocontrol agents and cultural practices that can contribute to *T. parvispinus* control. There is no doubt that conducting studies on bioecology and pest population dynamics is crucial for crafting an effective pest management and, in the long run, developing an integrated pest management program (IPM).

In response to the quarantine imposed to ornamental growers, we investigated insecticides with a knock-down effect. In the 2023 spring shipping season, more than 60 nurseries were placed under quarantine, but they were given the option of re-inspection within 48 h. Our work followed this short timeline and presents an initial set of insecticide alternatives to help growers and prevent further losses in the nurseries. However, this is only a first step in controlling the damage and expansion of this invasive and regulated pest, as more research is needed. Long-term experiments and greenhouse testing for the efficacy of these products will further contribute to validate the performance of these products over extended periods. By addressing these multifaceted aspects, we can ensure that the developmental path of pest control alternatives not only preserves effective pest control but also promotes sustainable agricultural practices and minimizes environmental impacts. 

## Figures and Tables

**Table 1:** List of conventional and biorational insecticides tested against *Thrips parvispinus* in laboratory experiments.

	Trade Name	Active Ingredient(s)	Insecticide Group ^a^	Rate ^b^	Rate in 1 L Solution	Site ^c^	EPA Registration Number ^d^
Conventional Insecticides	Acephate 97 UP WDG	Acephate	1B	91.8 g/ha	599 mg	G, N, L	70506-8
	Altus	Flupyradifurone	4D	167.6 mL/ha	1.09 mL	G, N, L	432-1575
	Aria	Flonicamid	29	33.3 g/ha	211 mg	G, N, L	279-3287
	AzaSol	Azadirachtin	Unknown	68.8 g/ha	898 mg	G, N, I, L	81899-4-74578
	Conserve SC	Spinosad	5	1.20 mL/ha	0.78 mL	G, N, L	62719-291
	Fulcrum	Pyriproxyfen	7C	143.6 mL/ha	0.94 mL	G, N, L, S	59807-14
	Hachi-Hachi SC	Tolfenpyrad	21A	323.1 mL/ha	2.11 mL	G, N, S, L	71711-31-67690
	Kontos	Spirotetramat	23	40.7 mL/ha	0.26 mL	G, N, I	432-1471
	Mainspring GNL	Cyantraniliprole	28	95.7 mL/ha	0.63 mL	G, N, I, L	10015-43
	Merit 75 WSP	Imidacloprid	4A	18 g/ha	37 mg	N, L, I	432-1318
	Overture 35 WP	Pyridalyl	Unclassified	91.8 g/ha	599 mg	G	59639-125
	Pedestal	Novaluron	15	95.7 mL/ha	0.63 mL	G, N, S	53883-419-59807
	Piston TR	Chlorfenapyr	13	119.7 mL/ha	0.78 mL	G	91234-19
	Pradia	Cyclaniliprole-Flonicamid	28–29	209.4 mL/ha	1.37 mL	G, N, S	71512-33-59807
	Rycar	Pyrifluquinazon	9B	38.3 mL/ha	0.25 mL	G	71711-37-67690
	Sarisa	Cyclaniliprole	28	323.1 mL/ha	2.11 mL	G, N, S	71512-32-59807
	Sevin SL	Carbaryl	1A	383 mL/ha	2.5 mL	G, N, L	432-1227
	Talstar P	Bifenthrin	3A	259.7 mL/ha	1.70 mL	G, N, L	279-3206
	Timectin 0.15 EC	Abamectin	6	95.7 mL/ha	0.63 mL	S, G, N	84229-1
	Tristar 8.5 SL	Acetamiprid	4A	302.8 mL/ha	1.98 mL	G, N, S, L	8033-106-1001
	Xxpire	Sulfoxaflor-Spinetoram	4C-5	31.5 g/ha	206 mg	G, N	62719-676
Biorational Insecticides	Agropest	Thyme + rosemary oil	Unclassified	0.5%	5 mL	S, G, N, L	FIFRA 25 (b) exempt
	Arte + Guard	Artemisia afra + canola oil	Unclassified	12.0 mL/ha	7.81 mL	G, N, I, L	FIFRA 25 (b) exempt
	Bee Safe 3-in-1	Sesame oil	Unclassified	35.9 mL/ha	23.02 mL	S, G, N, L	FIFRA 25 (b) exempt
	Bush Doctor Force of Nature	Garlic oil	Unclassified	1531.9 mL/ha	9.99 mL	S, G, N, L	FIFRA 25 (b) exempt
	M-Pede	Potassium salts of fatty acids	Unclassified	29.9 mL/ha	20.07 mL	G, N, L, I	10163-324
	Nuke EM	Citric acid	Unclassified	95.7 mL/ha	62.5 mL	S, G, N, L	FIFRA 25 (b) exempt
	Sierra Natural Science 209	Rosemary oil	Unclassified	646.3 mL/ha	8.44 mL	S, G, N, S	FIFRA 25 (b) exempt
	Stylet-Oil JMS	Paraffinic oil	Unclassified	12.0 mL/ha	7.81 mL	G, N, I, L	65564-1
	SuffOil-X	Mineral oil	Unclassified	2%	20 mL	G, N, L	48813-1-68539
	Thyme Guard	Thyme oil	Unclassified	0.5%	5 mL	S, G, N, L	FIFRA 25 (b) exempt
	Ultra-fine	Mineral oil	Unclassified	3%	30 mL	G, N, L, I	86330-11

^a^ Insecticide group is based on the IRAC mode of action classification. ^b^ Rate calculations are based on the label-recommended amount of product to be applied to one hectare (ha). ^c^ Recommended application site for each product (S: shadehouse, G: greenhouse, N: nursery, L: landscape, I: interior). ^d^ All products are registered and commercially available for use in Florida against thrips on ornamental plants.

## Data Availability

The datasets generated and analyzed during the current study are available in the figshare repository under the https://doi.org/10.6084/m9.figshare.24961707 (accessed on 10 November 2023).
